# Supplementary figures and images for: The Cellular NMD Pathway Restricts Zika Virus Infection and Is Targeted by the Viral Capsid Protein
Source: mBio. 2018 Nov 6;9(6):e02126-18. doi: 10.1128/mBio.02126-18 (PMC6222128; doi:10.1128/mBio.02126-18)

Supplementary Figure 1

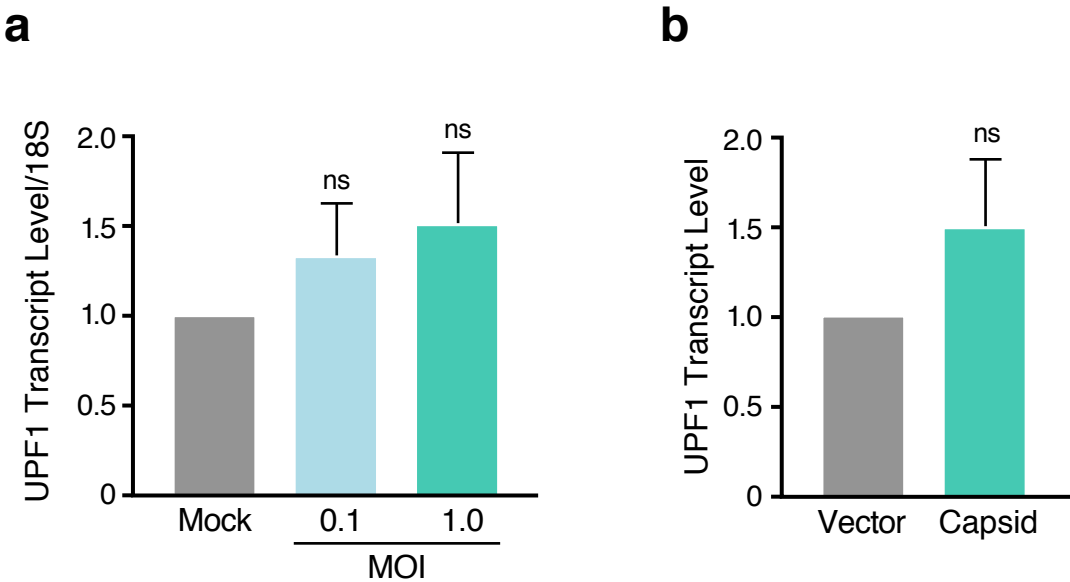

Supplement: FIG S1 [file mbo005184151sf1.pdf]

Supplementary Figure 2

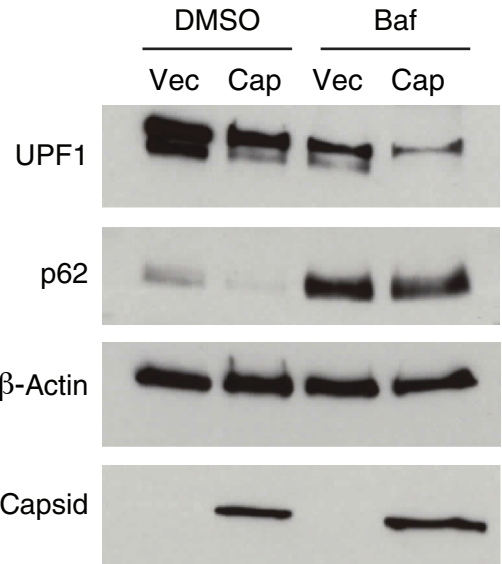

Supplement: FIG S2 [file mbo005184151sf2.pdf]

Supplementary Figure 3

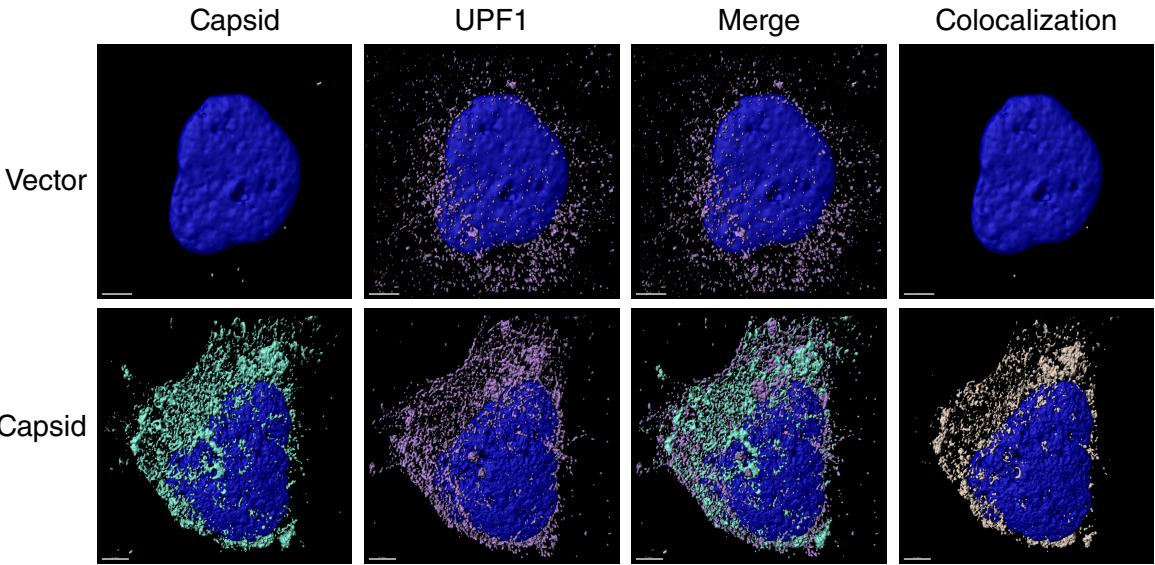

Supplement: FIG S3 [file mbo005184151sf3.pdf]
